# IL‐8 from CD248‐expressing cancer‐associated fibroblasts generates cisplatin resistance in non‐small cell lung cancer

**DOI:** 10.1111/jcmm.18185

**Published:** 2024-02-23

**Authors:** Jieheng Wu, Qiaoling Zhang, Jiangwei Wu, Zeyang Yang, Xinlei Liu, Chunju Lou, Xuanyin Wang, Jiangying Peng, Jinyuan Zhang, Zhenling Shang, Jing Xiao, Nianxue Wang, Ruya Zhang, Jinyao Zhou, Yun Wang, Zuquan Hu, Rui Zhang, Jian Zhang, Zhu Zeng

**Affiliations:** ^1^ Department of Immunology Guizhou Medical University Guiyang Guizhou China; ^2^ The State Key Laboratory of Cancer Biology, Department of Biochemistry and Molecular Biology The Fourth Military Medical University Xi'an China; ^3^ Key Laboratory of Infectious Immune and Antibody Engineering of Guizhou Province, Engineering Research Center of Cellular Immunotherapy of Guizhou Province, School of Biology and Engineering Guizhou Medical University Guiyang Guizhou China; ^4^ Key Laboratory of Biology and Medical Engineering, Immune Cells and Antibody Engineering Research Center of Guizhou Province Guizhou Medical University Guiyang Guizhou China; ^5^ Guizhou Prenatal Diagnsis Center The Affiliated Hospital of Guizhou Medical University Guiyang Guizhou China; ^6^ Department of Pharmaceutical analysis Zunyi Medical University Zunyi Guizhou China; ^7^ School of Health Management Guangzhou Medical University Guangzhou Guangdong China; ^8^ Department of Thoracic Surgery The Affiliated Hospital of Guizhou Medical University Guiyang Guizhou China

**Keywords:** cancer‐associated fibroblasts, CD248, chemoresistance, IL‐8, non‐small cell lung cancer

## Abstract

Chemotherapy‐resistant non‐small cell lung cancer (NSCLC) presents a substantial barrier to effective care. It is still unclear how cancer‐associated fibroblasts (CAFs) contribute to NSCLC resistance to chemotherapy. Here, we found that CD248^+^CAFs released IL‐8 in NSCLC, which, in turn, enhanced the cisplatin (CDDP) IC50 in A549 and NCI‐H460 while decreasing the apoptotic percentage of A549 and NCI‐H460 in vitro. The CD248^+^CAFs‐based IL‐8 secretion induced NSCLC chemoresistance by stimulating nuclear factor kappa B (NF‐κB) and elevating ATP‐binding cassette transporter B1 (ABCB1). We also revealed that the CD248^+^CAFs‐based IL‐8 release enhanced cisplatin chemoresistance in NSCLC mouse models in vivo. Relative to wild‐type control mice, the CD248 conditional knockout mice exhibited significant reduction of IL‐8 secretion, which, in turn, enhanced the therapeutic efficacy of cisplatin in vivo. In summary, our study identified CD248 activates the NF‐κB axis, which, consecutively induces the CAFs‐based secretion of IL‐8, which promotes NSCLC chemoresistance. This report highlights a potential new approach to enhancing the chemotherapeutic potential of NSCLC‐treating cisplatin.

## 1 INTRODUCTION

Lung cancer (LC) is a relatively common form of malignancy. In fact, among all malignant tumours, LC ranks the second and first place in terms of incidence and mortality rates. Non‐small cell LC (NSCLC) is a highly prevalent LC type, accounting for almost 80%–85% of all new LC incidences.^1^ Currently, in addition to surgical intervention, radiotherapy and traditional chemotherapy, NSCLC is also treated with immune checkpoint therapy and small molecule inhibitors that target mutation sites of LC cell signalling pathways. Despite continuous advancements in chemo‐and molecular targeted therapies, the 5‐year and total survival rate of NSCLC patients remain exceedingly poor. The primary reason for this is that NSCLC is resistant to chemotherapy.^2^, ^3^ Hence, exploring the molecular mechanisms associated with NSCLC chemical resistance has great potential in enhancing patient outcome and overall survival (OS).

Chemoresistance is an unavoidable challenge of tumour therapy. Hence, tumour chemoresistance is a major focus of cancer research. Tumour cells can directly or indirectly develop resistance to chemical drugs using strategies like tumour cell heterogeneity, augmented DNA repair, enhanced drug efflux, up‐regulated angiogenesis, tumour metabolic alterations, changes in tumour cell genetics epigenetic modifications and changes in the tumour microenvironment (TME).^4^

Cancer‐associated fibroblasts (CAFs) are crucial constituents of the TME. They physically interact with numerous immune cells to accelerate tumour development and progression. This is done by remodelling the TME, enhancing tumour angiogenesis, and sculpting the extracellular matrix (ECM).^5^, ^6^, ^7^

More recently, it was demonstrated that the TME positively modulates tumour cell chemoresistance, in addition to the regulatory alterations within tumour cells themselves. CAFs form the major constituents of TME, and they are intricately linked to the induction of tumour cell chemoresistance.^8^ The CAF‐driven tumour chemoresistance can be divided into two categories: via cell adhesion mediated drug resistance (CAM‐DR), and via release of soluble and secretory factors mediated drug resistance (SFM‐DR). The CAM‐DR‐induced drug resistance utilizes proteoglycans on the CAFs surface to adhere to matrix fibroblasts or ECM components. Alternately, SFM‐DR utilizes cytokine release by CAFs to accelerate tumour cell chemoresistance.^9^ Nevertheless, the associated mechanisms underlying the CAFs‐mediated NSCLC chemoresistance remains poorly elucidated.

CD248, otherwise called endosialin or tumour endothelial marker 1(TEM1), does not typically express in healthy tissues, and is ubiquitous in stromal cells, such as, activated fibroblasts, tumour perivascular cells or during inflammatory diseases.^10^ Emerging evidences highlight CD248 as a particular bioindicator of activated fibroblasts, and this includes CAFs in tumours.^11^ In our pervious study, we demonstrated that CD248 is ubiquitous within CAFs from NSCLC tissues, and its expression is intricately linked to worse outcome and clinicopathological profiles, namely, NSCLC‐related tumour‐node metastasis (TNM) stage, lymphatic metastasis and tumour stage and differentiation.^12^

Herein, our analyses revealed that CD248^+^CAFs release IL‐8 in NSCLC, which, in turn, promoted cisplatin chemoresistance in NSCLC in vivo and in vitro. Based on these functional investigations, CD248 modulated the nuclear factor kappa B (NF‐κB) axis to enhance the CAFs‐mediated release of IL‐8, which resulted in NSCLC cisplatin resistance, and additionally, activated NF‐κB and up‐regulated ATP‐binding cassette transporter B1 (ABCB1) expression. Herein, we highlighted the relevance and associated signalling network of CD248^+^CAFs in NSCLC chemoresistance, and presented a new approach of enhancing the chemotherapeutic efficacy within NSCLC patients.

## 2 MATERIALS AND METHODS

### 2.1 Human tumour specimen extraction

The Affiliated Hospital of Guizhou Medical University provided biopsies of human NSCLC as well as matched healthy tissues for comparison. All participants provided written informed permission before the research began, and the study was authorized by the same institution's ethics committee (approval no. 2022LL‐49).

### 2.2 Mice

Mice with floxed *cd248* or *fsp‐1*‐Cre were established by Suzhou Cyagen Co., Ltd. The *fsp‐1*‐Cre mice were cross‐bred with floxed *cd248* mice to develop *cd248* ^fl/fl^ *fsp‐1* ^+/+^ (WT), and CD248 fibroblasts conditional knockout mice *cd248* ^fl/fl^ *fsp‐1* ^cre/+^ (cKO). All mice were maintained in specific pathogen‐free environments, with 12‐h light/dark cycle and room temperature (RT) and humidity adjusted to 22 ± 1°C and 55% ± 5%., respectively. The animals received standard laboratory chow. All experimental mice originated from a C57BL/6 genetic background, were housed in separate cages, and were experimented on between the ages of 6–12 weeks. Cre‐negative littermate were housed together, and served as controls for all experiments. Our animal protocol received ethical approval from the Guizhou Medical University.

### 2.3 Cell lines and coculture assay

Short tandem repeat (STR) analysis confirmed the absence of Mycoplasma contamination in the human NSCLC cell lines A549 and NCI‐H460 and the murine LC cell line Lewis lung carcinoma (LLC1), both of which were obtained from the American Type Culture Collection (ATCC, Manassas, VA, USA). Cells were maintained in Roswell Park Memorial Institute (RPMI) 1640 medium or Dulbecco's Modified Eagle Medium/Nutrient Mixture F‐12 (DMEM/F12) (Gibco Life Technologies, Waltham, MA, USA) with 10% FBS (Gibco Life Technologies, Waltham, MA, USA) and 1% penicillin–streptomycin (Invitrogen Life Technologies, Waltham, MA, USA) at 37°C in a 5% CO_2_ humid chamber. Fibroblast extraction and cultivation were carried out according to the protocol of our prior investigation.^12^, ^13^ We created CAFs‐sh‐CON cells by infecting them with control lentivirus, created CAFs‐sh‐CD248 cells with stable CD248 suppression and also created CAFs‐CD248OE cells with overexpression CD248 by infecting them with lentivirus.^12^

### 2.4 Coculture experiments

To conduct co‐culture experiments, we plated A549 and NCI‐H460 cells into six‐well plates in conditioned medium (CM) from CAFs, CAFs‐sh‐CON or CAFs‐sh‐CD248, prior to a 48‐h cisplatin (MedChemExpress, #HY‐17394) exposure. The cells were analysed upon reaching 90% confluency.

### 2.5 Cytokine arrays

We employed a Proteome Profiler Human XL Cytokine Array Kit (R&D Systems, #ARY022B, Minneapolis, MN, USA), as directed in associated protocol. After 1 h of blocking in Human Cytokine Array Detection Antibodies Combination at RT, membranes were treated overnight (ON) at 4°C with 200 μg of CAFs, CAFs‐sh‐CON or CAFs‐sh‐CD248 overall protein. All membranes were twice rinsed, before a 30‐min exposure to HRP‐conjugated streptavidin at RT. Before treating the membranes with a chemiluminescent substrate and taking images of them, we gave them a second rinsing. Finally, we used the MultiImage Light Cabinet Filtering Settings from the Alpha Innotech Corporation in Santa Clara, California.

### 2.6 Western blot

The employed primary antibodies are as follows: anti‐α‐smooth muscle actin(anti‐α‐SMA) (Servicebio, #GB111364), anti‐human CD248 (CST, #47948, Louisville, KY, USA), anti‐AKT (CST, #9272), anti‐Phospho‐Akt (Ser473) (CST, #4060), anti‐Phospho‐PI3 Kinase p85 (Tyr458)/p55(Tyr199) (CST, #4228), anti‐PI3 Kinase p85 (CST, #4257), anti‐ABCB1 (CST, #13342), anti‐NF‐κB Pathway Antibody Sampler Kit (CST, #9936), anti‐IL‐8 (CST, #94407) and anti‐β‐actin (Servicebio, #GB15003). MultiImage was utilized for visualizing protein after being separated total protein lysate in sodium dodecyl sulfate‐polyacrylamide gel electrophoresis (SDS‐PAGE) using electrophoresis, prior to transfer to polyvinylidene difluoride membranes, which were then blocked for 2 h at RT in 5% bovine serum albumin (BSA) in Tris‐Buffered Saline with Tween (TBST), prior to treatment with any of the aforementioned primary antibodies and correspond horseradish peroxidase (HRP)‐conjugated antibody (Servicebio, #GB23303; #GB23301).

### 2.7 Enzyme‐linked immunosorbent assay (ELISA)

CAFs‐sh‐CON and CAFs‐sh‐CD248 were grown in fresh serum‐free media for 24 h. Subsequently, the culture media was assays for IL‐8 expression using corresponding ELISA kit (Absin, #abs510004) and associated directions.

### 2.8 Immunofluorescence (IF) and immunohistochemistry (IHC) stainings

The primary antibodies used in IHC and IF evaluations are anti‐CD31 (CST, #3528), anti‐α‐SMA (Abcam, #ab5694), anti‐human CD248 (CST, #47948), anti‐p65 (CST, #8242), TUNEL Apoptosis Assay Kit (Beyotime, #C1089), Ki‐67 (Abcam, #ab16667) and anti‐IL‐8 (Absin, #abs136518). To conduct IHC assessment, slides underwent blocking in endogenous peroxidase, prior to a 30‐min treatment with goat serum at RT, with subsequent ON incubation at 4°C in anti‐CD248 antibody (1:150), Ki‐67 (1:100) or anti‐IL‐8 antibody (1:100). Peroxidase activity was recorded using 3,3′‐diaminobenzidine (DAB), and image capture was done under a microscope.

To conduct TUNEL evaluation, the slides underwent a 20‐min treatment at RT with Proteinase K, prior to a 1‐h incubation in TUNEL assay buffer at 37°C without light. Nuclear staining was done with diamidino‐2‐phenylindole (DAPI), and image capture with laser scanning confocal microscopy.

Six randomly chosen fields of view were examined per group at 400× magnification to determine the total number of Ki‐67 or TUNEL‐positive cells. Mean ± standard deviation (SDs) expressed data.

Tyramide signal amplification (TSA) was utilized for conducting IF evaluation for detecting α‐SMA, CD248, CD31 and IL‐8 expressions and colocalizations in tumour tissue sections. In short, slides were placed for 15 min in 3% H_2_O_2_ at RT, prior to a 2‐h blocking in 5% BSA at RT, followed by an ON primary antibody treatment at 4°C (step 1), TBST rinsing, then a 50‐min incubation in HRP‐conjugated secondary antibody at RT (step 2). After exposing the TSA fluorophores to RT for 10 min (step 3), the antibody‐TSA complex was removed by subjecting the sample to a boiling cycle in ethylenediaminetetraacetic acid (EDTA) antigen retrieval solution (step 4). The first four procedures were repeated until every slide had been stained with every antibody. Lastly, we performed DAPI nuclear staining, then image capture under a laser scanning confocal microscope.

Before IF, cells were fixed in 4% paraformaldehyde for 15 min at RT, blocked in 5% BSA for 2 h at RT, then incubated with anti‐p65 (1:600) at 4°C ON and then treated with Alexa Fluor‐conjugated secondary antibodies (Servicebio) for 1 h at RT. Laser scanning confocal microscopy was utilized for image acquisition, while PI was employed to counterstain nuclei.

### 2.9 Real‐time quantitative polymerase chain reaction (RT‐qPCR)

MiniBEST Universal RNA Isolation Kit (Takara, #9767, San Jose, CA, USA) for total RNA isolation and PrimeScript RT Master Mix (Takara, #RR063A) for cDNA synthesis. Primers from Table S1 of the supplementary material were used in an RT‐qPCR performed with a TB Green *Premix Ex Taq* II Kit (Takara, #RR820A).

### 2.10 CCK‐8 evaluation

Cell survival was evaluated using Cell Counting Kit 8 (CCK‐8, Absin, #abs50003) assay. In short, 5 × 10^4^ cells were plated onto 96‐well plates and maintained ON at 37°C, before treatment with specified chemotherapeutic agents. Subsequently, we introduced CCK‐8 to the cells, followed by a 1.5‐h incubation at 37°C, then optical density measurement at 450 nm using CMax Plus (Molecular Devices). Six replicas were included per analysis, each experiment was repeated thrice.

### 2.11 Apoptosis evaluation

Cells underwent a 48‐h treatment with specified chemotherapeutic agents, prior to harvest via centrifugation. Apoptosis was assessed via FITC Annexin V Apoptosis Detection Kit (BD Biosciences, #550889, Haryana, India). In short, cells underwent a 15‐min treatment with 100 μL binding buffer (BB) plus 5 μL of FITC Annexin V and 5 μL PI at RT without light. Next, 400 μL BB was introduced, then apoptotic analysis was carried out by flow cytometry.

### 2.12 Mouse tumour experiments

Our animal protocols were ethically approved by Guizhou Medical University. We used 5 × 10^6^ A549 and 5 × 10^6^ CAFs cells infected with sh‐CON‐or sh‐CD248‐carrying lentiviruses, to generate the tumour cell and CAFs mixed models in BALB/c nude mice (4–6 weeks, male, body weight 18–20 g). For establishing the CD248 fibroblasts cKO mouse model, we subcutaneously administered 1 × 10^6^ LLC1 cells into the back of age‐ and sex‐matched WT and cKO mice. Following tumour development, mice were twice intravenously administered with cisplatin (2 mg/kg). The tumour volume was computed as follows: *V* (mm^3^) = a × b^2^/2, where a and b indicated the long and short diameters, respectively. After the trial period had ended, the animals were killed, and tumours were removed and analysed using imaging technology. Some tumour specimens could be utilized for protein extraction for the detection of CD248, IL‐8, and ABCB expressions. The remaining tumour tissues were paraffin embedding for IHC (IL‐8, CD248, TUNEL, and Ki‐67) together with staining via haematoxylin and eosin (H&E).

### 2.13 Statistical analyses

Data are expressed as mean ± SD. Two‐sided *t*‐tests (Microsoft excel 2016) was employed for *p*‐value determination. Quantitative data were assessed via normality test and equality test of variances. Lastly, *p <* 0.05 values were denoted as significant.

## 3 RESULTS

### 3.1 CD248‐expressing CAFs promoted cisplatin resistance in NSCLC

**FIGURE 1:**
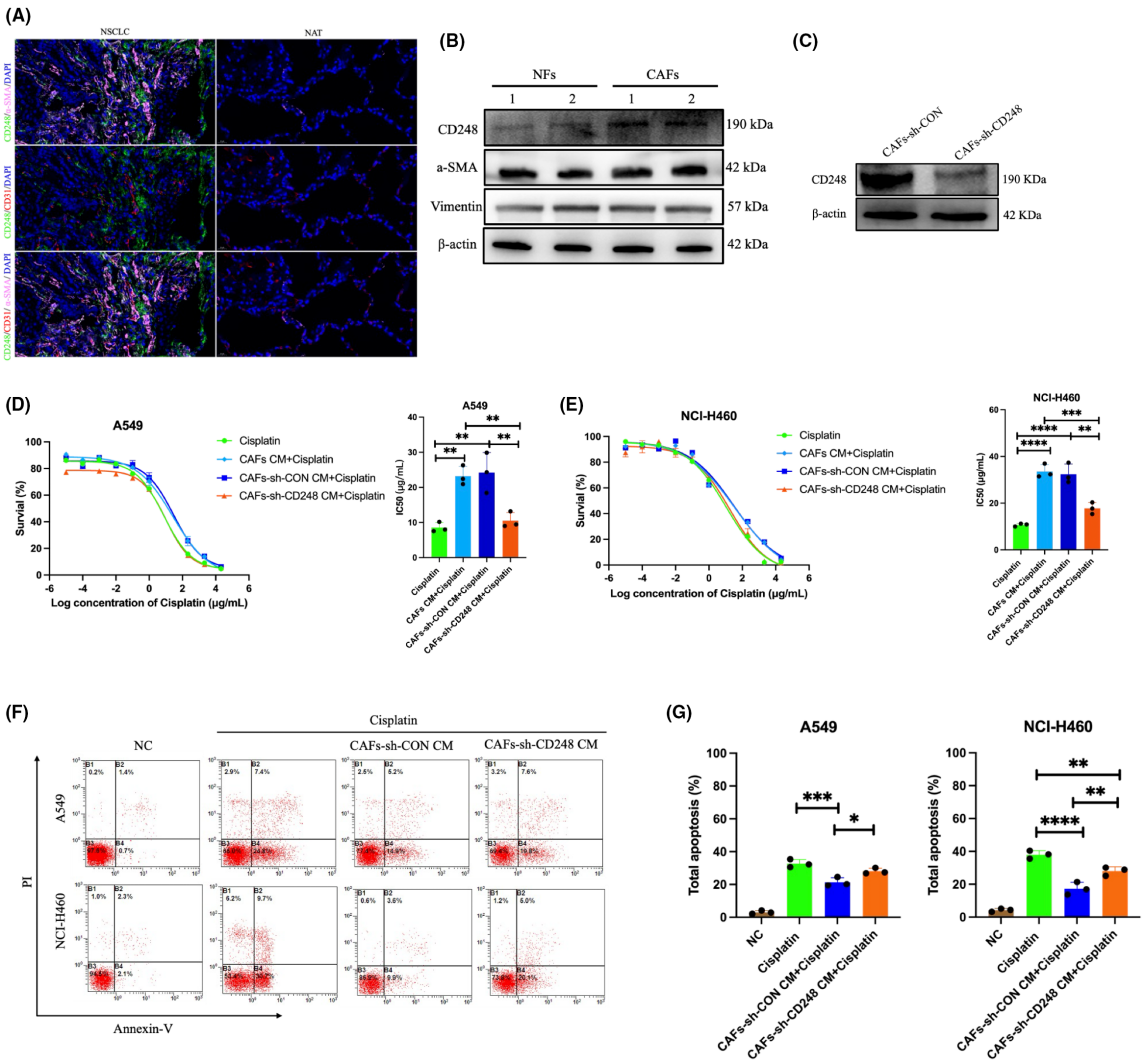
CD248‐expressing CAFs inhibited cisplatin killing of NSCLC. (A) Typical dual immunofluorescence (IF) images illustrating the α‐SMA, CD31 and CD248 colocalization within NATs and NSCLC samples. Evaluation of different biomarkers, including CD248 expressions within (B**)** extracted NFs and CAFs using western blotting and (C) CD248 expression in CAFs or CAFs‐sh‐CD248 using western blot. (D, E) Evaluation of cell survival following cisplatin treatment of A549 and NCI‐H460 cells cultured either alone (−) or with CM from CAFs‐sh‐CON/CAFs‐sh‐CD248, and computation of the IC50 concentration per group. All experiments were performed thrice. Data provided as mean ± SEM. (F) Apoptosis evaluation of cisplatin‐treated cells that were either cultured alone (−) or with CM from CAFs‐sh‐CON/CAFs‐sh‐CD248. (G) Evaluation and quantification of Annexin V^+^/PI^−^ (early apoptosis) and Annexin V^+^/PI^+^ (late apoptosis) cells. All experiments were conducted thrice. Mean ± SEM, *, *p <* 0.05, **, *p <* 0.01, ***, *p <* 0.001, ****, *p <* 0.0001. Scale bar, 20 μm.

We conducted IF analysis on NSCLC and nonneoplastic tissues to evaluate the CD248 expression profile in NSCLC. Figure 1A illustrates that CD248 was ubiquitously expressed in NSCLC tissues, and displayed little to no expression in nonneoplastic tissues. More specifically, CD248 exhibited a CAFs‐specific expression, as is evidenced by its colocalization with α‐SMA, and not with CD31 (Figure 1A). Next, we extracted fibroblasts from the tumour tissues (designated as CAFs) and adjoining healthy tissues (designated as NFs) of NSCLC patients. A closer examination of the CAFs and NFs revealed that both expressed Vimentin and α‐SMA, however, only CAFs displayed a significant proportion of CD248 expression (Figure 1B). In a prior investigation, we established a CAFs model via a stable knock down of CD248 expression. We further confirmed CD248 deficiency using western blot analysis (Figure 1C).

To elucidate whether the CD248‐expressing CAFs mediates cisplatin resistance in NSCLC, we treated NSCLC cell lines with CM from CAFs, CAFs‐sh‐CON or CD248‐sh‐CD248, prior to exposure to varying concentrations of cisplatin. Based on our findings, both A549 and NCI‐H460 cells exposed to CAF‐CM exhibited enhanced cisplatin IC50, relative to cisplatin‐treated cells. Alternately, the A549 and NCI‐H460 cells maintained in CAFs‐sh‐CD248 CM showed diminished cisplatin IC50, relative to CAFs or CAFs‐sh‐CON CM‐treated cells (*p <* 0.001) (Figure 1D,E). Using flow cytometry, we further revealed that the A549 and NCI‐H460 cells exposed to CAFs CM experienced reduced apoptosis, compared to cisplatin‐or CAFs‐sh‐CD248‐treated cells (*p <* 0.05) (Figure 1F,G). These findings indicated that the CD248‐expressing CAFs enhanced cisplatin resistance to NSCLC.

### 3.2 IL‐8 released by CD248‐expressing CAFs mediated cisplatin resistance in NSCLC

**FIGURE 2:**
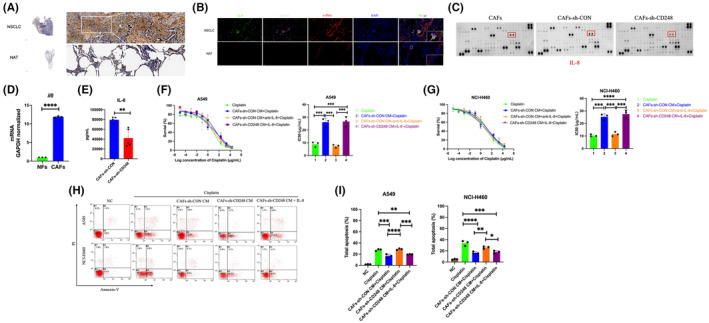
CD248‐expressing CAFs‐derived IL‐8 mediated chemoresistance. (A) Typical immunohistochemical (IHC) IL‐8 images from NSCLC biopsies and NATs (*n* = 50). (B) Typical dual immunofluorescence (IF) images illustrating the α‐SMA, IL‐8, and CD248 colocalization within NATs and NSCLC samples. Evaluation of IL‐8 secretion from CAFs‐sh‐CD248 or control CAFs (C) as evidenced by cytokine array, (D) qPCR, and (E) ELISA. All experiments were conducted in five replicas. Data provided as mean ± SEM. A two‐tailed *t*‐test designated *p*‐values. **, *p <* 0.01. (F, G) Evaluation of cell survival following cisplatin treatment of A549 and NCI‐H460 cells cultured either alone (−) or with CM from CAFs‐sh‐CON/CAFs‐sh‐CD248 or IL‐8, and computation of IC50 concentration per group. (H) Apoptosis evaluation of cisplatin‐treated cells cultured either alone (−) or with CM from CAFs‐sh‐CON/CAFs‐sh‐CD248 or IL‐8. (I), Evaluation and quantification of Annexin V^+^/PI^−^ (early apoptosis) and Annexin V^+^/PI^+^ (late apoptosis) cells. Frequency of experiments, data presentation and significance as above. Scale bar, 100 μm.

IL‐8 is a potent proinflammatory cytokine capable of inducing cancer cell chemoresistance. To examine whether CD248^+^CAFs mediates cisplatin chemoresistance in NSCLC through its release of IL‐8, we conducted IHC‐based IL‐8 evaluations within NSCLC and nonneoplastic tissues. According to our result, IL‐8 was ubiquitously present in NSCLC tissues, however, its expression was minimal in nonneoplastic tissues (Figure 2A). We further employed IF to assess IL‐8 presence within CD248^+^CAFs. We demonstrated that IL‐8 was strongly present among NSCLC‐related CD248^+^CAFs since it was colonized by α‐SMA and CD248 (Figure 2B). To explore whether CD248 influences CAFs‐mediated IL‐8 release, cytokine release alteration detection in CAFs‐sh‐CD248 was approached. We revealed that IL‐8 release was increased in the CAFs and CAFs‐sh‐CON than CAFs‐sh‐CD248 cells (Figure 2C). RT‐qPCR for IL‐8 expression assessment in CAFs‐sh‐CD248 was also approached. The IL‐8 content was strongly (*p <* 0.01) reduced within CAFs‐sh‐CD248 (Figure 2D). Subsequently, using ELISA, the CM from CAFs‐sh‐CD248 had markedly reduced IL‐8 expression, relative to CM from control CAFs, thus confirming the significance of CD248 in IL‐8 secretion by CAFs (*p <* 0.01) (Figure 2E).

To elucidate the underlying mechanism behind the IL‐8‐triggered chemoresistance, we examined IL‐8‐ or IL‐8 blockade antibody‐treated NSCLC cell lines, which were subsequently treated with CM from CAFs‐sh‐CD248 or control CAFs. Our results revealed that the A549 and NCI‐H460 cells that were maintained in CM from CAFs‐sh‐CD248 that were pre‐treated with IL‐8 exhibited enhanced cisplatin IC50. However, with IL‐8 blockade, the cisplatin IC50 diminished in both A549 and NCI‐H460 cell lines (*p <* 0.001) (Figure 2F,G). Using flow cytometry, we revealed that the A549 and NCI‐H460 cell apoptosis was enhanced in the IL‐8 blockade‐treated cells, while the apoptosis was reduced in cells maintained in CM from CAFs‐sh‐CD248 but were pre‐treated with IL‐8 (*p <* 0.05) (Figure 2H,I). Based on these results, IL‐8 enhances chemoresistance of NSCLC cells.

### 3.3 IL‐8 mediated cisplatin chemoresistance in human NSCLC cells by activating NF‐κB and enhancing ABCB1

**FIGURE 3:**
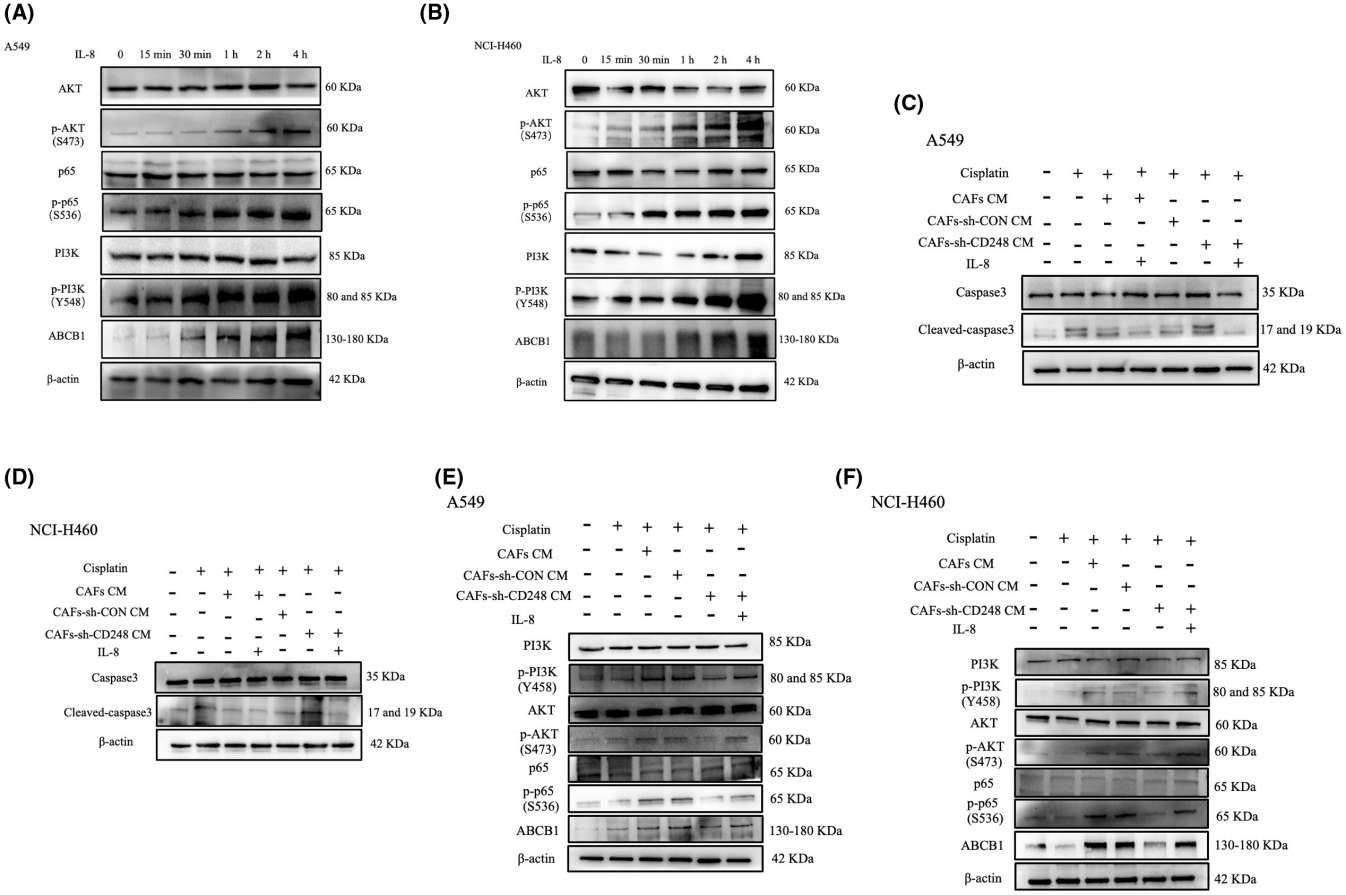
IL‐8 induced cisplatin chemoresistance in NSCLC cells by activating NF‐κB and up‐regulating ABCB1. (A) Evaluation of NF‐κB axis‐related and ABCB1 transcript expressions in cisplatin‐exposed A549 cells that were either cultured alone or with 100 ng/mL IL‐8 at varying time points, as evidenced by western blot. (B) Evaluation of NF‐κB axis‐related and ABCB1 protein expression in cisplatin‐treated NCI‐H460 cells that were either cultured alone or with 100 ng/mL IL‐8 at varying time points, as detected by western blot. (C) Evaluation of cleaved/total caspase‐3 in cisplatin‐treated A549 cells that were either cultured alone or with specified component. (D) Evaluation of cleaved/total caspase‐3 in cisplatin‐treated NCI‐H460 cells that were either cultured alone or with specified component. (E) Evaluation of the NF‐κB axis‐related and ABCB1 expression in cisplatin‐treated A549 cells that were either cultured alone or with specified CAFs. (F) Evaluation of the NF‐κB axis‐related and ABCB1 expressions in cisplatin‐treated NCI‐H460 cells that were either cultured alone or with specified CAFs, as detected by western blot.

ABCB1 serves an essential function as a drug pump to minimize intracellular drug content. However, its unchecked action can result in chemoresistance in multiple forms of human cells.^14^, ^15^ It was previously revealed that CAFs secrete IL‐8 to promote ABCB1 expression, which, in turn, enhances chemoresistance in gastric cancer cells via activation of the NF‐κB axis.^16^ To explore whether IL‐8 induces ABCB1 content via NF‐κB axis stimulation in NSCLC, we assessed the NF‐κB axis‐related protein expressions in IL‐8‐exposed NSCLC cells. We demonstrated that the ABCB1, p‐PI3K, p‐AKT, and p‐p65 contents were markedly augmented in both IL‐8‐treated A549 and NCI‐H460 cells (Figure 3A,B). Our results indicated that IL‐8 enhanced ABCB1 content via activation of NF‐κB.

To next explore whether IL‐8 induces cisplatin chemoresistance in NSCLC, we evaluated cleaved/total caspase‐3 expression in cisplatin‐exposed A549 and NCI‐H460 cells cultured either alone or with specified component using western blot assay. Based on our analyses, CM from CD248^+^CAFs or from CAFs‐sh‐CD248 with IL‐8 supplementation, and not from CAFs‐sh‐CD248, robustly protected cancer cell lines from chemotherapy‐triggered apoptosis (Figure 3C,D). To further examine whether CD248^+^CAFs induces cisplatin chemoresistance via NF‐κB/ABCB1, we next assessed the ABCB1 and NF‐κB axis‐related protein expression profile in both A549 and NCI‐H460 cells treated with CM from CAFs‐sh‐CD248 with IL‐8 or not or control CAFs. Our findings revealed that both ABCB1 and p‐p65 were present in excess in A549 and NCI‐H460 cells conditioned with CM from CAFs‐sh‐CD248 with IL‐8 and control CAFs (Figure 3E,F). Given these evidences, it is clear that CD248^+^CAFs‐released IL‐8 enhances cisplatin chemoresistance in NSCLC via activation of NF‐κB and elevation of ABCB1 expression.

### 3.4 CD248 induced CAFs‐mediated IL‐8 secretion via NF‐κB activation

**FIGURE 4:**
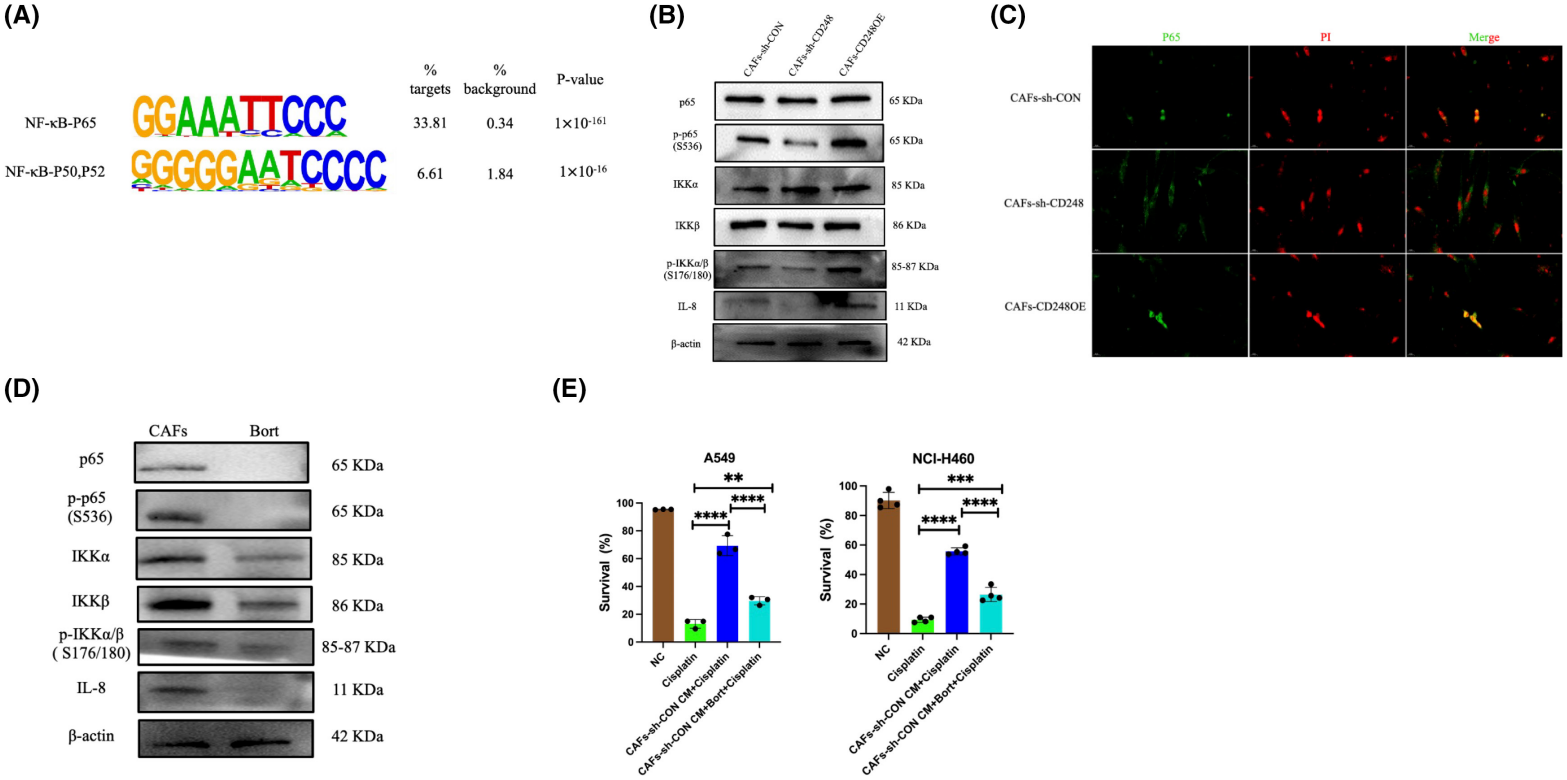
CD248 induced CAFs‐based IL‐8 secretion utilized NF‐κB activation. (A) Transcription factor binding motifs enriched in CD248^+^CAFs open chromatin regions using ATAC‐seq. (B) Evaluation of the expression of NF‐κB axis in CAFs‐sh‐CD248, CAFs‐sh‐CON, and CAFs‐CD248OE. (C) Immunofluorescent p65 (green) stain depicting the nuclear transfer of NF‐κB in CAFs‐sh‐CD248, CAFs‐sh‐CON, and CAFs‐CD248OE. Scale bar; 20 μm. (D) Evaluation of the NF‐κB axis‐related proteins in 4 nM Bortezomib‐treated CAFs, as evidenced by western blot. (E) Cell survival evaluation of cisplatin‐treated A549 and NCI‐H460 cells that were either cultured alone (−) or with CM from CAFs‐sh‐CD248/CAFs‐sh‐CON+Bortezomib. All experiments were conducted thrice. Data provided as mean ± SEM. **, *p <* 0.01, ***, *p <* 0.001, ****, *p <* 0.0001.

The NF‐κB axis is a prototypical proinflammatory network that activates proinflammatory genes, namely, cytokines, chemokines and adhesion molecules.^17^, ^18^ Using transposase‐accessible chromatin with high‐throughput sequencing (ATAC‐seq) assay, we revealed that the CD248^+^CAFs open chromatin regions were abundant in docking regions for the transcription factor p65 (Figure 4A). To validate whether CD248 activates NF‐κB, we employed western blot for the detection of NF‐κB axis‐related proteins in the CAFs‐sh‐CON, CAFs‐sh‐CD248 or CAFs‐CD248OE cells. We revealed that the CD248 accelerated a rise in cytoplasmic p‐IKKβ and nuclear p‐p65 protein contents (Figure 4B). Using IF staining, we further verified that CD248 overexpression enabled p65 nuclear transfer (Figure 4C). Moreover, we explored whether CD248 mediates CAFs IL‐8 secretion via activation of the NF‐κB axis. We treated CAFs with Bortezomib, a potent NF‐κB inhibitor that blocks IκB phosphorylation and NF‐κB nuclear transfer, thereby diminishing expression of downstream cytokines. We demonstrated that Bortezomib strongly suppressed the CD248‐driven rise in cytoplasmic p‐IKKβ, nuclear p‐p65 and IL‐8 protein expressions (Figure 4D). Subsequently, we treated the NSCLC cell lines with CM from CAFs or Bortezomib‐treated CAFs to assess its in vitro influence on IL‐8 in response to cisplatin treatment. We revealed that the A549 and NCI‐H460 cells exposed to CM from Bortezomib‐treated CAFs exhibited reduced survival, relative to those conditioned in CM from untreated CAFs (*p <* 0.0001) (Figure 4E). Taken together, our findings suggested that CD248 enhanced the CAFs‐mediated release of IL‐8 via NF‐κB activation.

### 3.5 CD248‐expressing CAFs facilitated IL‐8 secretion, which mediated cisplatin chemoresistance in NSCLC in vivo

**FIGURE 5:**
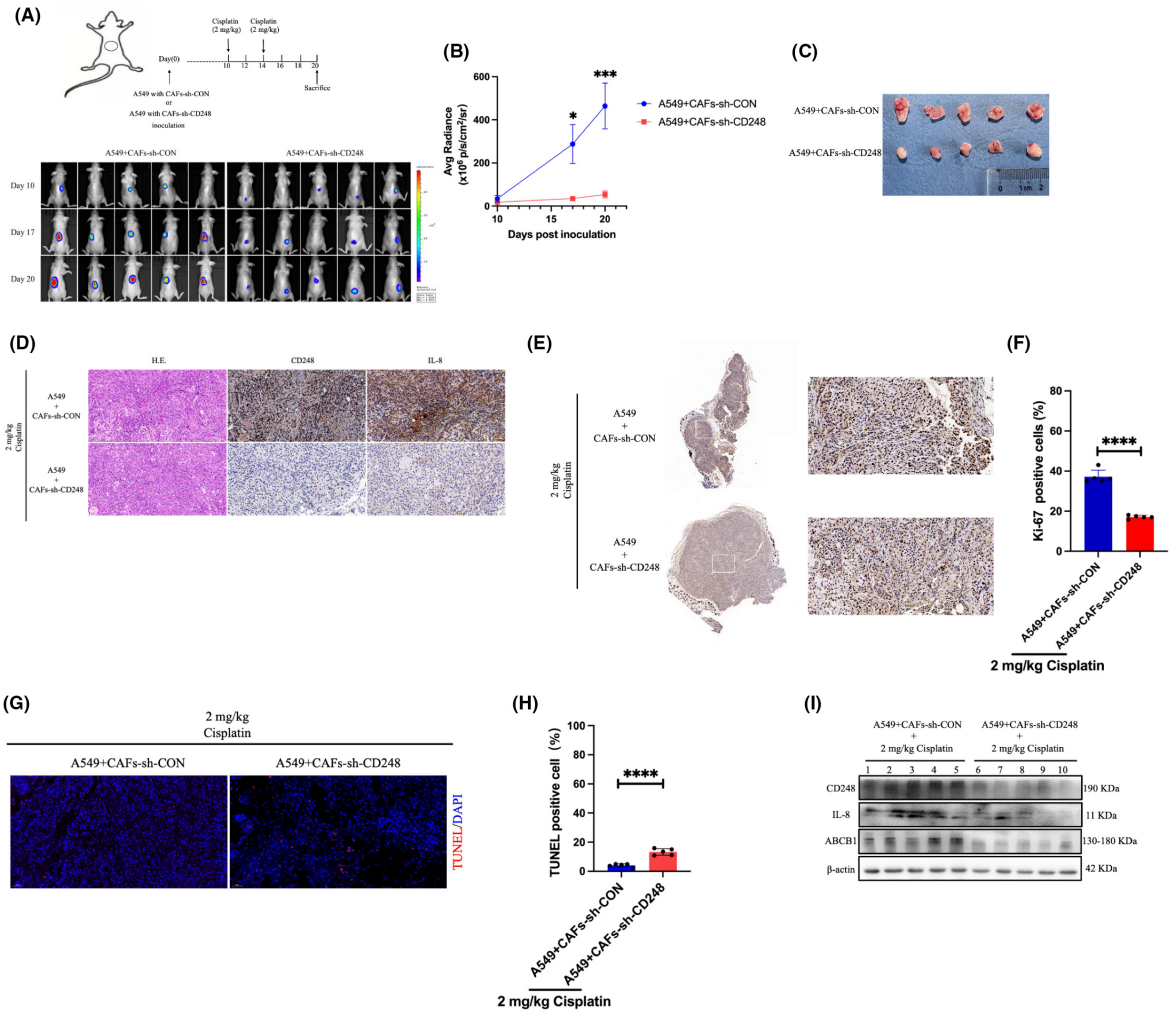
CD248‐positive CAFs mediated NSCLC cisplatin resistance in vivo. (A) The mouse NSCLC model utilizing A549 cells, together with control CAFs and CAFs‐sh‐CD248 xenografts, is shown schematically. When CAFs‐sh‐CD248 and A549 or control CAFs (*n* = 5 mice/group) were injected into mice, tumour development was monitored by BLI. (B) Fluorescence intensity assessment per group. *, *p <* 0.05, ***, *p <* 0.001. (C) Tumour dissection and photography at 19 days post inoculation. (D) IL‐8 and CD248 evaluations using IHC and H&E staining. (E) Ki‐67 staining of tumour tissue sections for cell proliferation assessment. (F) Quantification of Ki‐67–positive cells. (G) TUNEL (red) staining illustrating cell apoptosis in tumour tissues. (H)TUNEL–positive cells quantification. (I) Evaluation of CD248, ABCB1, and IL‐8 expressions in the examined xenografts (*n* = 5 mice per group). Significance and presentation of data as before. Scale bar, 50 μm.

To elucidate whether CAFs containing CD248 release IL‐8, which, in turn, promote cisplatin chemoresistance in NSCLC in vivo, we first combined 5 × 10^6^ A549 and 5 × 10^6^ CAFs cells infected with sh‐CON‐or sh‐CD248‐carrying lentiviruses, then we administered the aforementioned cells into BALB/c nude mice, and then injected 2 mg/kg cisplatin into the A549 + CAFs‐sh‐CD248 or A549 + CAFs‐sh‐CON mice. We carefully monitored tumour development with bioluminescence imaging (BLI), and recorded the tumour fluorescence intensity. Based on our findings, tumour development was strongly (*p <* 0.05) enhanced by the A549 and CAFs‐sh‐CON cell combination, relative to the A549 and CAFs‐sh‐CD248 cell combination (Figure 5A–C). Furthermore, H&E findings revealed that the established tumour tissues were NSCLC (Figure 5D). Based on our IHC CD248 and IL‐8 stainings, we revealed that both proteins were abundantly expressed in A549 + CAFs‐sh‐CON mice versus A549 + CAFs‐sh‐CD248 mice (Figure 5D). Ki‐67‐positive cell quantity was markedly reduced among the A549 and CAFs‐sh‐CD248 cells, relative to the CD248‐expressing CAFs cells, based on Ki‐67 IHC staining, thereby indicating that the cisplatin therapeutic activity was significantly inhibited in the CAFs‐sh‐CD248 xenografts (*p <* 0.001) (Figure 5E,F). Lastly, using IF TUNEL staining, we revealed that the cisplatin‐treated CAFs‐sh‐CD248 xenografts displayed reduced apoptosis, relative to the cisplatin‐treated A549 + CAFs‐sh‐CON mice (Figure 5G,H). Subsequently, we conducted western blot analysis to assess the contents of CD248, ABCB1 and IL‐8 proteins. We revealed that the aforementioned proteins were strongly diminished in the cisplatin‐treated CAFs‐sh‐CD248 xenografts, relative to the cisplatin‐treated A549 + CAFs‐sh‐CON mice (Figure 5I). Collectively, these evidences suggested that the CD248‐expressing CAFs secreted IL‐8, which, in turn, promoted cisplatin chemoresistance in NSCLC in vivo.

### 3.6 Fibroblasts‐specific *CD248* depletion promoted cisplatin killing NSCLC in vivo

**FIGURE 6:**
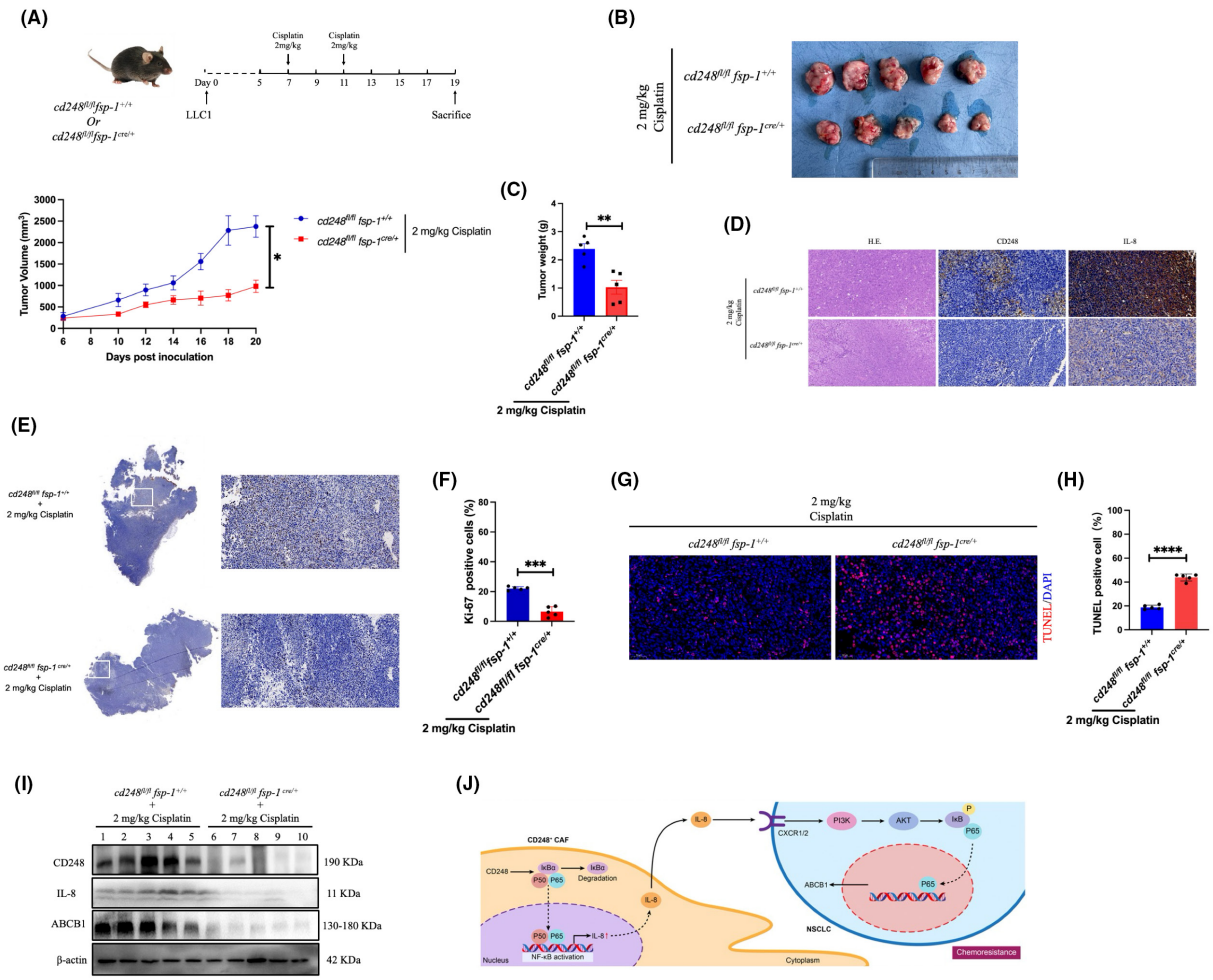
Fibroblast‐specific CD248 deletion in mice prevented lung cancer (LC) chemoresistance. We subcutaneously administered 5 × 10^6^ LLC1 cells into *cd248* ^*fl/fl*^ *fsp‐1* ^*+/+*^ (WT) and *cd248* ^*fl/fl*^ *fsp‐1* ^*cre/+*^(cKO) mice to develop our xenografts. (A) A schematic depicting our mouse model. Evaluation of tumour development following cisplatin treatment, using tumour volume analysis. (B) Tumour dissection and photography at 19 days post inoculation. (C) Quantification of tumour volume. (D) Typical IHC images of IL‐8 and CD248, as well as H&E images. (E) Ki‐67‐stained tumour tissue sections doe cell proliferation assessment. (F) Quantification of Ki‐67–positive cells. (G) TUNEL (red) staining depicting cell apoptosis in tumour tissues. (H) TUNEL–positive cells quantification. (I) Evaluation of the CD248, ABCB1, and IL‐8 protein expressions in the examined xenografts (*n* = 5 mice per group) as evidenced by western blot. (J) An illustration of CD248‐expressing CAFs‐derived IL‐8 mediating cisplatin resistance in NSCLC. Significance and presentation of data as before. Scale bar, 50 μm.

To characterize a possible CD248 involvement in NSCLC cisplatin chemoresistance, we injected cisplatin (2 mg/kg) into *CD248* ^*fl/fl*^ *fsp‐1* ^*+/+*^ (WT) or *CD248* ^*fl/fl*^ *fsp‐1* ^*cre/+*^ (cKO) mice with Lewis's xenografts, measured the tumour volume, and generated a tumour growth curve. We revealed that the tumour growth in conditional CD248 knockout mice was dramatically reduced, relative to WT mice (*p <* 0.05) (Figure 6A,B). We demonstrated that the tumour weight of conditional CD248 knockout mice was considerably less than the WT mice (*p <* 0.01) (Figure 6C). Our H&E staining revealed that the tumour tissues were NSCLC (Figure 6D). Using IHC CD248 and IL‐8 stainings, we revealed that both proteins were abundantly expressed in WT versus conditional CD248 knockout mice (Figure 6D). Ki‐67‐positive cell quantity was markedly (*p <* 0.001) reduced in the conditional CD248 knockout versus WT mice, based on Ki‐67 IHC staining, indicating that the tumour cell proliferation was markedly inhibited in conditional CD248 knockout mice (Figure 6E,F). Moreover, IF TUNEL evaluation demonstrated that the cisplatin‐treated conditional CD248 knockout mice had strongly reduced apoptosis, relative to cisplatin‐treated WT mice (Figure 6G,H). Lastly, using western blot analysis, we revealed that the CD248, ABCB1, and IL‐8 protein expressions were markedly diminished in the cisplatin‐treated conditional CD248 knockout mice, relative to cisplatin‐treated WT mice (Figure 6I). Collectively, our findings indicated that the CD248‐expressing CAFs secreted IL‐8, which, in turn, enhanced NSCLC cisplatin chemoresistance in vivo. Therefore, herein, we provided evidence that CD248^+^CAFs release IL‐8, which augment ABCB1 expression in NSCLC cell to enhance cisplatin resistance in NSCLC (Figure 6J).

## 4 DISCUSSION

The TME is composed of tumour and nontumor cells. Among the nontumor cells are fibroblasts, innate immune and adaptive immune cells.^8^ CAFs are crucial modulators of the TME, and they are known to induce tumour chemoresistance via release of cytokines and chemokines, and via augmentation of ECM remodelling.^19^

IL‐8, otherwise called CXCL8, is a critical cytokine that positively regulates neutrophil chemotaxis and inflammation.^20^, ^21^ It is secreted by numerous cells, namely, neutrophils, monocytes, endothelial cells and tumour cells. Emerging evidences suggested that the circulating IL‐8 levels in tumour patients is strongly associated with worse patient outcome and chemoresistance.^21^ CAFs are known to promote tumour cell chemoresistance by secreting IL‐8. It was reported that, in NSCLC, CAFs‐mediated IL‐8 secretion strongly suppress the therapeutic effect of fluorouracil (5‐FU) on tumour cells, while promoting strong chemoresistance.^22^ Several investigations reported that elevated IL‐8 expressions were present among patients with advanced primary gastric cancer, which, in turn, reduced the efficacies of neoadjuvant chemotherapy. Follow‐up investigations further confirmed that excess CAFs‐mediated secretion led to enhanced serum IL‐8 levels, which, in turn, activated the NF‐κB axis, thereby enhancing the tumour cell resistance gene ABCB1 expression, thus ultimately inducing cisplatin resistance against gastric cancer.^16^

Chemoresistance is typically mediated via the expression of drug‐related resistance genes. ATP binding cassette superfamily transporter (ABC) serves a crucial role in tumour chemoresistance. ABC primarily utilizes energy from ATP hydrolysis to modulate transmembranal transport of multiple substrates.^23^ Tumour cells generally express marked elevations in the ABC protein, which, in turn, translocate chemicals from tumour cells to ECM, thus inducing tumour chemoresistance. Among the ABC family members, ABCB1, otherwise called multidrug resistance protein 1 (MDR1), is highly significant.^24^, ^25^, ^26^ ABCB1 is ubiquitously present among LC, ovarian cancer, breast cancer, glioblastoma and renal cancer, and it is responsible for promoting tumour chemoresistance to drugs, such as, anthracycline actinomycin D, cisplatin, colchicine, etoposide, teniposide, methotrexate and others. One investigation revealed that CAFs enhance ABCB1 expression in gastric cancer cells via IL‐8 secretion, suppress cisplatin therapeutic effect, and enhance the occurrence and advancement of gastric cancer.^27^

CAFs are a heterogeneous population within the TME.^28^ They are composed of diver cell types that are sensitive to distinct stimuli within the TME. These variant cell types also display distinctive phenotypes, and take part in exclusive physiological functions. CD248 is typically present within tumour stromal fibroblasts and perivascular cells, and has minimal expression within healthy regions.^29^ Additionally, CD248 is overexpressed in multiple cancers, such as, breast and skin cancers, neuroblastomas and sarcomas.^30^ It is strongly associated with tumour angiogenesis and metastasis.^31^ Studies revealed that antibodies that block or knock out CD248 strongly suppress tumour cell development and angiogenesis.^31^, ^32^ Together, whether CD248 induces NSCLC chemoresistance requires further exploration.

In this report, we revealed that the CD248^+^CAFs released IL‐8 in NSCLC, which, in turn, enhanced cisplatin IC50, while decreasing apoptosis of A549 and NCI‐H460 cells in vitro. Next, we examined the associated signalling network(s) whereby CD248 enhances CAFs‐mediated IL‐8 secretion, which, in turn, induces NSCLC chemoresistance. We further utilized western blot and qRT‐PCR analyses to demonstrate that CD248^+^CAFs derived IL‐8 enhanced NSCLC chemoresistance via up‐regulation of ABCB1. Taken together, CD248 accelerates CAFs‐mediated secretion of IL‐8 by activating NF‐κB. Next, we validated that CD248‐expressing CAFs‐derived IL‐8 stimulates NSCLC cisplatin chemoresistance in vivo. Relative to WT mice, CD248 cKO mice exhibited reduced IL‐8 secretion, which, in turn, enhanced the therapeutic effect of cisplatin in vivo. This investigation highlights a new approach to enhancing the chemotherapeutical potential of anti‐NSCLC chemotherapy.

Briefly, herein, we demonstrated the importance of CD248 expression in CAFs, which enhance IL‐8 secretion, which, in turn, induce NSCLC chemoresistance. Our evidences also highlight CD248 as a novel bioindicator of NSCLC‐based CAFs, and both IL‐8 and CD248 as potential targets of anti‐NSCLC chemotherapy.

## AUTHOR CONTRIBUTIONS

**Jieheng Wu:** Data curation (lead); formal analysis (lead); funding acquisition (lead); investigation (lead); supervision (lead); writing – original draft (lead). **Qiaoling Zhang:** Data curation (equal); formal analysis (equal); methodology (equal); visualization (equal). **Jiangwei Wu:** Formal analysis (equal); investigation (equal); methodology (equal). **Zeyang Yang:** Investigation (equal); project administration (equal). **Xinlei liu:** Data curation (equal); project administration (equal); resources (equal). **Chunju Lou:** Data curation (supporting); investigation (supporting); project administration (supporting). **Xuanyin Wang:** Data curation (supporting); formal analysis (supporting). **Jiangying Peng:** Data curation (supporting); project administration (supporting); resources (supporting). **Jinyuan Zhang:** Data curation (supporting); formal analysis (supporting); methodology (supporting); visualization (supporting). **Zhenling Shang:** Project administration (supporting); resources (supporting); validation (supporting). **Jing Xiao:** Investigation (supporting); project administration (supporting). **Nianxue Wang:** Formal analysis (supporting); investigation (supporting); methodology (supporting). **Ruya Zhang:** Data curation (supporting); project administration (supporting). **Jinyao Zhou:** Formal analysis (supporting); investigation (supporting); methodology (supporting). **Yun Wang:** Data curation (supporting); project administration (supporting); resources (supporting). **Zuquan Hu:** Data curation (supporting); project administration (supporting). **Rui Zhang:** Data curation (supporting); project administration (supporting). **Jian Zhang:** Conceptualization (lead); data curation (equal); resources (lead); supervision (equal); visualization (equal); writing – review and editing (equal). **zhu Zeng:** Conceptualization (equal); data curation (equal); funding acquisition (equal); investigation (equal); methodology (equal); resources (equal); supervision (equal).

## FUNDING INFORMATION

This work was supported by the National Natural Science Foundation of China (Grant Nos. 82 160,566, 12 132 006), the Basic Research Program of Guizhou Provincial Department of Science and Technology Agency (ZK [2021]‐118), the National Natural Science Foundation of China Cultivation Program of Guizhou Medical University (21NSFCP26, 20NSP002), the Flexible Talent Introduction Project of Guizhou Medical University (RN2021‐GK045), and the High‐level Talent Startup Fund Project of Guizhou Medical University (J2020‐77).

## CONFLICT OF INTEREST STATEMENT

The authors declare no conflicts of interest.

## Supporting information


Table S1:

## Data Availability

The datasets used for the current study are available from the corresponding author on reasonable request.

## References

[1] Siegel RL , Miller KD , Fuchs HE , Jemal A . Cancer statistics, 2022. CA Cancer J Clin. 2022;72(1):7‐33. doi:10.3322/caac.21708 35020204

[2] Sedighzadeh SS , Khoshbin AP , Razi S , Keshavarz‐Fathi M , Rezaei N . A narrative review of tumor‐associated macrophages in lung cancer: regulation of macrophage polarization and therapeutic implications. Transl Lung Cancer Res. 2021;10(4):1889‐1916. doi:10.21037/tlcr-20-1241 34012800 PMC8107755

[3] Ben‐Baruch A . Inflammation‐associated immune suppression in cancer: the roles played by cytokines, chemokines and additional mediators. Semin Cancer Biol. 2006;16(1):38‐52. doi:10.1016/j.semcancer.2005.07.006 16139507

[4] Das CK , Mandal M , Kogel D . Pro‐survival autophagy and cancer cell resistance to therapy. Cancer Metastasis Rev. 2018;37(4):749‐766. doi:10.1007/s10555-018-9727-z 29536228

[5] Komohara Y , Takeya M . CAFs and TAMs: maestros of the tumour microenvironment. J Pathol. 2017;241(3):313‐315. doi:10.1002/path.4824 27753093

[6] Farhood B , Najafi M , Mortezaee K . Cancer‐associated fibroblasts: secretions, interactions, and therapy. J Cell Biochem. 2019;120(3):2791‐2800. doi:10.1002/jcb.27703 30260049

[7] Kalluri R . The biology and function of fibroblasts in cancer. Nat Rev Cancer. 2016;16(9):582‐598. doi:10.1038/nrc.2016.73 27550820

[8] Sahai E , Astsaturov I , Cukierman E , et al. A framework for advancing our understanding of cancer‐associated fibroblasts. Nat Rev Cancer. 2020;20(3):174‐186. doi:10.1038/s41568-019-0238-1 31980749 PMC7046529

[9] Meads MB , Gatenby RA , Dalton WS . Environment‐mediated drug resistance: a major contributor to minimal residual disease. Nat Rev Cancer. 2009;9(9):665‐674. doi:10.1038/nrc2714 19693095

[10] Xu C , Zhang K , Yang F , et al. CD248(+) cancer‐associated fibroblasts: a novel prognostic and therapeutic target for renal cell carcinoma. Front Oncol. 2021;11:773063. doi:10.3389/fonc.2021.773063 34970489 PMC8712640

[11] Matsushima S , Aoshima Y , Akamatsu T , et al. CD248 and integrin alpha‐8 are candidate markers for differentiating lung fibroblast subtypes. BMC Pulm Med. 2020;20(1):21. doi:10.1186/s12890-020-1054-9 31964365 PMC6975017

[12] Wu J , Liu X , Wu J , et al. CXCL12 derived from CD248‐expressing cancer‐associated fibroblasts mediates M2‐polarized macrophages to promote nonsmall cell lung cancer progression. Biochim Biophys Acta Mol Basis Dis. 2022;1868(11):166521. doi:10.1016/j.bbadis.2022.166521 35985448

[13] Yang F , Wei Y , Han D , et al. Interaction with CD68 and regulation of GAS6 expression by endosialin in fibroblasts drives recruitment and polarization of macrophages in hepatocellular carcinoma. Cancer Res. 2020;80(18):3892‐3905. doi:10.1158/0008-5472.CAN-19-2691 32591411

[14] Robey RW , Pluchino KM , Hall MD , Fojo AT , Bates SE , Gottesman MM . Revisiting the role of ABC transporters in multidrug‐resistant cancer. Nat Rev Cancer. 2018;18(7):452‐464. doi:10.1038/s41568-018-0005-8 29643473 PMC6622180

[15] Luo X , Teng QX , Dong JY , et al. Antimicrobial peptide reverses ABCB1‐mediated chemotherapeutic drug resistance. Front Pharmacol. 2020;11:1208. doi:10.3389/fphar.2020.01208 32903706 PMC7438908

[16] Zhai J , Shen J , Xie G , et al. Cancer‐associated fibroblasts‐derived IL‐8 mediates resistance to cisplatin in human gastric cancer. Cancer Lett. 2019;454:37‐43. doi:10.1016/j.canlet.2019.04.002 30978440

[17] Hayden MS , Ghosh S . Shared principles in NF‐kappaB signaling. Cell. 2008;132(3):344‐362. doi:10.1016/j.cell.2008.01.020 18267068

[18] Lawrence T . The nuclear factor NF‐kappaB pathway in inflammation. Cold Spring Harb Perspect Biol. 2009;1(6):a001651. doi:10.1101/cshperspect.a001651 20457564 PMC2882124

[19] Monteran L , Erez N . The dark side of fibroblasts: cancer‐associated fibroblasts as mediators of immunosuppression in the tumor microenvironment. Front Immunol. 2019;10:1835. doi:10.3389/fimmu.2019.01835 31428105 PMC6688105

[20] Wroblewski M , Szewczyk‐Golec K , Holynska‐Iwan I , Wroblewska J , Wozniak A . Characteristics of selected adipokines in ascites and blood of ovarian cancer patients. Cancers (Basel). 2021;13(18):4702. doi:10.3390/cancers13184702 34572929 PMC8465310

[21] Park SY , Han J , Kim JB , et al. Interleukin‐8 is related to poor chemotherapeutic response and tumourigenicity in hepatocellular carcinoma. Eur J Cancer. 2014;50(2):341‐350. doi:10.1016/j.ejca.2013.09.021 24161763

[22] Ding S , Tang Z , Jiang Y , et al. HDAC1 regulates the chemosensitivity of laryngeal carcinoma cells via modulation of interleukin‐8 expression. Eur J Pharmacol. 2021;896:173923. doi:10.1016/j.ejphar.2021.173923 33539818

[23] Begicevic RR , Falasca M . ABC transporters in cancer stem cells: beyond chemoresistance. Int J Mol Sci. 2017;18(11):2362. doi:10.3390/ijms18112362 29117122 PMC5713331

[24] Xi G , Hayes E , Lewis R , et al. CD133 and DNA‐PK regulate MDR1 via the PI3K‐or Akt‐NF‐κB pathway in multidrug‐resistant glioblastoma cells in vitro. Oncogene. 2016;35(42):5576. doi:10.1038/onc.2016.64 27593924

[25] Huang B , Fu SJ , Fan WZ , et al. PKCepsilon inhibits isolation and stemness of side population cells via the suppression of ABCB1 transporter and PI3K/Akt, MAPK/ERK signaling in renal cell carcinoma cell line 769P. Cancer Lett. 2016;376(1):148‐154. doi:10.1016/j.canlet.2016.03.041 27037060

[26] Shaffer BC , Gillet JP , Patel C , Baer MR , Bates SE , Gottesman MM . Drug resistance: still a daunting challenge to the successful treatment of AML. Drug Resist Updat. 2012;15(1‐2):62‐69. doi:10.1016/j.drup.2012.02.001 22409994 PMC3348380

[27] Barbato L , Bocchetti M , Di Biase A , Regad T . Cancer stem cells and targeting strategies. Cell. 2019;8(8):926‐945. doi:10.3390/cells8080926 PMC6721823 31426611

[28] Wang Z . CAF heterogeneity and dynamics. Nat Cell Biol. 2022;24(12):1686. doi:10.1038/s41556-022-01054-z 36474074

[29] Teicher BA . CD248: a therapeutic target in cancer and fibrotic diseases. Oncotarget. 2019;10(9):993‐1009. doi:10.18632/oncotarget.26590 30847027 PMC6398180

[30] Rouleau C , Curiel M , Weber W , et al. Endosialin protein expression and therapeutic target potential in human solid tumors: sarcoma versus carcinoma. Clin Cancer Res. 2008;14(22):7223‐7236. doi:10.1158/1078-0432.CCR-08-0499 19010839

[31] Li C , Wang J , Hu J , et al. Development, optimization, and validation of novel anti‐TEM1/CD248 affinity agent for optical imaging in cancer. Oncotarget. 2014;5(16):6994‐7012. doi:10.18632/oncotarget.2188 25051365 PMC4196179

[32] Li C , Chacko AM , Hu J , et al. Antibody‐based tumor vascular theranostics targeting endosialin/TEM1 in a new mouse tumor vascular model. Cancer Biol Ther. 2014;15(4):443‐451. doi:10.4161/cbt.27825 24553243 PMC3979822

