# Supplementary material for: IL‐8 from CD248‐expressing cancer‐associated fibroblasts generates cisplatin resistance in non‐small cell lung cancer
Source: J Cell Mol Med. 2024 Feb 23;28(4):e18185. doi: 10.1111/jcmm.18185 (PMC10891307; doi:10.1111/jcmm.18185)
Supplement: Supplementary file 1 — Table S1: [file JCMM-28-e18185-s001.docx]

**Supplementary Table S1**

**Primer sequence for q-PCR**

| gene name | Forward primer: | Reverse primer: |
| --- | --- | --- |
| Human  *il8* | 5’-ACACTGCGCCAACACAGAAATTA-3’ | 5’- TTTGCTTGAAGTTTCACTGGCATC-3’ |
| Human GAPDH | 5’-GGAGCGAGATCCCTCCAAAAT-3’ | 5’-GGCTGTTGTCATACTTCTCATGG-3’ |
